# Role of Vitamin C in Skin Diseases

**DOI:** 10.3389/fphys.2018.00819

**Published:** 2018-07-04

**Authors:** Kaiqin Wang, Hui Jiang, Wenshuang Li, Mingyue Qiang, Tianxiang Dong, Hongbin Li

**Affiliations:** ^1^Department of Dermatology, First Affiliated Hospital of Kunming Medical University, Kunming, China; ^2^Bio-ID Center, School of Biomedical Engineering, Shanghai Jiao Tong University, Shanghai, China

**Keywords:** vitamin C, atopic dermatitis, porphyria cutanea tarda, malignant melanoma, herpes zoster

## Abstract

Vitamin C (ascorbic acid) plays an important role in maintaining skin health and can promote the differentiation of keratinocytes and decrease melanin synthesis, leading to antioxidant protection against UV-induced photodamage. Normal skin needs high concentrations of vitamin C, which plays many roles in the skin, including the formation of the skin barrier and collagen in the dermis, the ability to counteract skin oxidation, and the modulation of cell signal pathways of cell growth and differentiation. However, vitamin C deficiency can cause or aggravate the occurrence and development of some skin diseases, such as atopic dermatitis (AD) and porphyria cutanea tarda (PCT). Levels of vitamin C in plasma are decreased in AD, and vitamin C deficiency may be one of the factors that contributes to the pathogenesis of PCT. On the other hand, high doses of vitamin C have significantly reduced cancer cell viability, as well as invasiveness, and induced apoptosis in human malignant melanoma. In this review, we will summarize the effects of vitamin C on four skin diseases (porphyria cutanea tarda, atopic dermatitis, malignant melanoma, and herpes zoster and postherpetic neuralgia) and highlight the potential of vitamin C as a therapeutic strategy to treat these diseases, emphasizing the clinical application of vitamin C as an adjuvant for drugs or physical therapy in other skin diseases.

## The Physiology of Vitamin C in Skin

Vitamin C (ascorbic acid, ascorbate) is a simple low-molecular-weight carbohydrate that is essential for the body as a water-soluble vitamin (Lykkesfeldt et al., 2014). As an antioxidant, vitamin C has both oxidized and reduced forms in the body: L-dehydroascorbic and L-ascorbic acid. Although vitamin C is an important antioxidant, humans and other primates obtain vitamin C only from their diet, because they have no ability to synthesize it. With blood circulation to all tissues and organs, plasma ascorbate acid concentrations can reach up to 10–160 mM (1–15 mg/ml) after eating a vitamin C diet, and the superfluous vitamin can be excreted by the kidneys (Richelle et al., 2009). However, there are large differences in the levels of vitamin C in various organs; for example, the brain, liver, and skeletal muscle have the highest total content, and the content of testis and thyroid is low (Omaye et al., 1987).

The skin is the largest multifunctional organ on the surface of the human body and consists of three layers: the epidermis, dermis, and subcutaneous tissue, which forms a complete whole with tension and elasticity as the body’s first line of defense against harmful external factors (Hunter, 1973). The epidermis is composed of keratinocytes and dendritic cells, and the stratum corneum can prevent both harmful substances and skin moisture loss and is evolved from keratinocytes and its lipid matrix (Tagami, 2008); the dermis provides nutrition for the skin and is rich in blood vessels and nerve endings (Rittie and Fisher, 2015); and the connective tissue is composed of collagen fibers and elastic fibers in the dermis, which maintains the tension and elasticity of the skin (Carl and Enna, 1979). There is a large difference in the content of vitamin C in the layers of the skin. The content of ascorbic acid in the epidermis is 425% higher than the content in the dermis, and there is a concentration gradient of ascorbic acid in the epidermal keratinocytes (Shindo et al., 1994; Weber et al., 1999).

**FIGURE 1:**
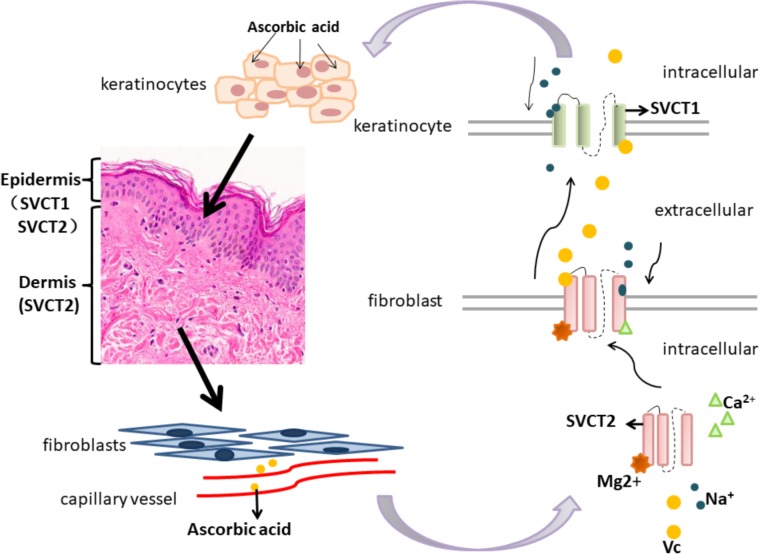
Vitamin C transporters (SVCT1 and SVCT2) and their transport mechanisms in skin.

It is well known that there are two transport mechanisms for ascorbic acid in the skin, and they depend on sodium-ascorbate cotransporter-1 (SVCT1) and sodium-ascorbate cotransporter-2 (SVCT2). Dermal fibroblasts present two high-affinity and low-affinity vitamin C transport mechanisms, which may be related to plasma concentrations of ascorbic acid or stress conditions (Butler et al., 1991), demonstrating that skin vitamin C transport characteristics may be associated with skin healing, antioxidation, and antitumor effects. Sodium-ascorbate cotransporters (SVCTs), specific sodium-dependent vitamin C transporters, exist in various tissues and organs for vitamin C uptake and transport. SVCT1 is primarily responsible for the transport of epidermal vitamin C, while SVCT2 is responsible for intradermal transport, both of which are shown in **Figure 1**. SVCT2 in dermal cells (such as fibroblasts) diffuses ascorbic acid transported from the plasma into the epidermis, and SVCT1 in the epidermis supplies ascorbic acid to keratinocytes (Steiling et al., 2007). The SVCT2 transporter in fibroblasts in the dermis transports vitamin C from the blood into the cells (Steiling et al., 2007). If SVCT2 is inside the fibroblasts, it can bind to Mg^2+^ but is in a low-affinity state. On the other hand, when SVCT2 is exposed on the fibroblast membrane surface, it can bind to both Mg^2+^ and Ca^2+^ in high concentrations of sodium solution and then becomes a high-affinity state and binds to vitamin C (Savini et al., 2008). Vitamin C can be transported into the cell after binding to SVCT1 on the membrane of keratinocytes, and vitamin C and Na^+^ are reversed on the cell membrane at a 1:2 ratio and then discretely distributed in epidermal keratinocytes (Wang et al., 2000; Steiling et al., 2007; Savini et al., 2008). The expression of SVCT1 mRNA in mouse skin under UVB irradiation showed time- and dose-dependent effects, whereas the SVCT2 mRNA levels did not change significantly, which seems to explain why the antioxidant capacity of the epidermis is greater than that of the dermis (Kang et al., 2007).

## The Role of Vitamin C in Skin

Vitamin C is involved in the formation of the skin barrier and collagen in the dermis and plays a physiological role in the skin against skin oxidation, in antiaging of wrinkles, and in cell signal pathways of cell growth and differentiation, which are related to the occurrence and development of various skin diseases (Ponec et al., 1997b). Vitamin C has a dual role of antioxidation and pro-oxidation, and this role maintains the balance of the two reactions in the body (Kim K. et al., 2015). Ascorbic acid and transition metals, such as Fe^2+^, produce reactive oxygen species (ROS) outside of the cell, and high levels of ROS can destroy the antioxidant defense system of cancer cells (Ohno et al., 2009; Conner et al., 2012) because the antioxidation system of tumor cells is incomplete and its balance is destroyed (Kim K. et al., 2015; Uetaki et al., 2015). High levels of Vitamin C in the cells lead to oxygen-promoting reactions, which cause DNA damage, the depletion of ATP reserves, and failure of cellular metabolism (Tian et al., 2014). Vitamin C is also involved in resistance to UV-induced oxidative stress, inhibition of melanogenesis, and promotion of the differentiation of keratinocytes and has been used for a long time as a clinical treatment reagent. Vitamin C deficiency leads to many systemic diseases in humans and causes scurvy in the world’s navies (Carpenter, 2012).

### Resistance to UV-Induced Oxidative Stress

Ultraviolet light, especially UVA, is an important factor that induces skin oxidative stress (McArdle et al., 2002). UVA radiation of the skin produces pyrimidine dimers and singlet oxygen in the body. The former weakens the hydrogen bonding effects between DNA double strands. The latter can generate the entire oxygen radical cascade and leads to the alteration of nucleic acids, proteins and lipids, which may induce skin tumors (Lin et al., 2005; Rinnerthaler et al., 2015) There is a sophisticated and complete antioxidant system in the skin, which is used as a defense to the oxidation reaction induced by UV or ozone. The antioxidant system consists of two categories, including the enzyme antioxidant system [superoxide dismutase (SOD) and catalase (CAT)] and non-enzymatic antioxidant system (vitamin C, vitamin E, and glutathione). The accumulation of ROS over the range of antioxidant defenses leads to skin diseases (Godic et al., 2014). However, vitamin C as a supplement has its own instability. Moreover, topical vitamin C supplementation can counteract oxidative stress induced by UVA, which can be assessed in human skin by the chemiluminescence method (Ou-Yang et al., 2004). In addition, the mRNA expression level of matrix metalloproteinase-1 (MMP-1) is significantly increased in the dermal fibroblast after UVA irradiation (Offord et al., 2002). Here, vitamin C may prevent collagen degradation and inhibit the increase of MMP-1, which is the major collagenolytic enzyme responsible for collagen damage in UV-irradiated skin (Offord et al., 2002; Brennan et al., 2003). Moreover, the combination of vitamin E, vitamin C, and ferulic acid can reduce the incidence of oxidative stress-induced tumors, and their antioxidant effects are much better than the use of vitamin C alone (Lin et al., 2005).

### Inhibiting Melanogenesis

The synthesis of melanin occurs in the melanocytes of the basal layer of the epidermis and can be transferred to keratinocytes so that melanin is distributed throughout the epidermis (Kwak et al., 2015). Tyrosine and 2-hydroxyphenylalanine (L-dopa) are oxidized to melanin by tyrosinase, which is the rate-limiting enzyme in the whole process (Bin et al., 2014). Whether vitamin C can inhibit melanogenesis is controversial. Most studies have agreed that although it cannot kill melanocytes, vitamin C does inhibit melanogenesis; however, some investigators have demonstrated that the role of vitamin C in the inhibition of melanogenesis is very weak and cannot inhibit tyrosinase activity (Shimada et al., 2009; Panich et al., 2011). Furthermore, the combination of vitamin C and vitamin E inhibits melanocyte production more significantly than vitamin C alone (Choi et al., 2010a).

### Promoting Differentiation of Keratinocytes

The integrity of the skin barrier may be related to the differentiation of keratinocytes, which affects the function of the skin barrier and causes skin diseases. Vitamin C enhances the late differentiation of keratinocytes, overcomes the differentiation-dependent oxidative stress, and maintains the integrity of the entire cuticle (Ponec et al., 1997a; Savini et al., 2002), which is an important prerequisite for the integrity of the skin barrier, ensuring the function of the skin barrier and preventing skin water loss, which in turn can lead to skin disorders.

## Vitamin C-Related Skin Diseases

The effects of food restriction on changes in nutrient intake and severity of the skin disease have been investigated; with ascorbate as a prodrug in various skin diseases, clinical treatment strategies for how to correctly apply vitamin C have become of interest to many dermatologists. Herein, we summarize the application of vitamin C as a treatment for a variety of skin diseases, such as porphyria cutanea tarda (PCT), atopic dermatitis (AD), malignant melanoma, herpes zoster (HZ), and postherpetic neuralgia (PHN), in clinic according to well-known levels of evidence (**Table 1**).

**Table 1:** The roles of vitamin C in skin disease.

Skin disease	Pathogenesis	Clinical lesions feature	The roles of vitamin C	Reference
Porphyria cutanea tarda (PCT)	Plasma ascorbate deficiency, protoporphyrin decarboxylase activity in urine and the accumulation of uroporphyrin in the liver	Acute and chronic blistering of the skin exposed to sunlight	Inhibit the catalytic oxidation of CYP1LA2, reduce the accumulation of urinary porphyrins in the liver	Percy et al., 1975; Monteiro et al., 1989; Sinclair et al., 1993, 1995, 1997a,b; Thunell et al., 1995; Anderson, 2007; Gorman et al., 2007; Ferrer et al., 2010; Ferrer et al., 2013; Patil et al., 2016
Atopic dermatitis (AD)	Structural or functional damage of the skin barrier	Erythematous papules with itching or scaling	Promote keratinocyte differentiation and the production of interstitial material, maintain skin barrier function	Cook et al., 1997; Forastiere et al., 2000; Uchida et al., 2001; Arora et al., 2002; Savini et al., 2002; Bieber, 2008; Kim et al., 2011, 2013; Kim K.P. et al., 2015; Assier et al., 2013; Lim et al., 2013; Sivaranjani, 2013; Park and Zippin, 2014; Tollefson and Bruckner, 2014; Shin et al., 2016; Zaniboni et al., 2016
Malignant melanoma (MM)	Gene mutation, oxidative stress, epigenetic changes, tumor microenvironment, etc.	Blue-black or brown papules and nodules, partially papillomatous, and verrucous-like lesions with ulcers	Inhibiting the HIF-1α transcriptional activity, increasing 5hm content in melanoma cells and maintaining tumor capsule integrity can prevent tumor invasion and metastasis	Kameyama et al., 1996; Feskanich et al., 2003; Bedogni and Powell, 2009; Ohno et al., 2009; Choi et al., 2010b; Cha et al., 2011; Levine et al., 2011; Cha et al., 2013; Stojkovic-Filipovic and Kittler, 2014; Hill et al., 2015; Kaminska-Winciorek et al., 2015; Gustafson et al., 2015; Kim K. et al., 2015; Miles et al., 2015; Uetaki et al., 2015; Yang et al., 2017; Yussif et al., 2017
Herpes zoster (HZ) and Postherpetic Neuralgia (PHN)	Disinhibition, central sensitization, reactive oxygen species (ROS), and neuroinflammation	Clustered small blisters distributed along the unilateral peripheral nerves with acute neuralgia.	Reduce pain and prevent the onset of PHN	Kim et al., 2004, 2016; Insinga et al., 2005; Weaver, 2009; Schencking et al., 2010; Byun and Jeon, 2011; Chen et al., 2011; Nalamachu and Morley-Forster, 2012; Gan et al., 2013; Nair et al., 2014; Chao et al., 2015; Carr and McCall, 2017; Hemilä, 2017; Marrero et al., 2017


### Porphyria Cutanea Tarda

Variegate porphyria (VP) is an autosomal dominant type of hepatic porphyria. Women affected by VP appear with chronic inflammation, plasma oxidative damage and decreased protoporphyrinogen oxidase (PPOX), CAT and glutathione reductase (GR) activities that make heme precursors chronically accumulate in erythrocytes, possibly inducing cellular damage (Monteiro et al., 1989; Thunell et al., 1995; Ferrer et al., 2013). PCT is a common type of porphyria in humans and is characterized clinically by acute and chronic blistering of the skin when exposed to sunlight, which usually occurs in the middle and late stages of the disease and causes great suffering among patients (Patil et al., 2016). The main role of uroporphyrin deacidification enzyme is the carboxylation of uroporphyrinogen (Sinclair et al., 1993, 1995). Cytochrome p450, especially cytochrome P450 1A2 (CYP1A2), can catalyze the oxidation of uroporphyrins to form uroporphyrins (Sinclair et al., 1995, 1997b). PCT can be caused by a decrease in protoporphyrin decarboxylase activity in urine and the accumulation of uroporphyrin in the liver. Plasma ascorbate deficiency may be a factor that leads to PCT, and a deficiency in ascorbic acid plays roles in some patients with PCT (Percy et al., 1975; Sinclair et al., 1997a; Anderson, 2007). Ascorbic acid as an antioxidant can inhibit the catalytic oxidation of CYP1LA2. Thus, vitamin C is a good potential drug for the treatment of PCT. The occurrence and development of the disease are related to the iron content: the greater the iron load, the more severe the disease (Sinclair et al., 1995). On the other hand, ascorbic acid can inhibit the accumulation of URO at low concentrations of iron, but the effect of ascorbic acid at high concentrations of iron (300–500 mg Fe/kg) is offset (Gorman et al., 2007). Ascorbic acid promotes iron absorption in the intestine, which may be risky in patients with iron overload. Therefore, solving for the iron inhibition of ascorbic acid is the main problem associated with using ascorbic acid as a clinical treatment. Oral supplementation with vitamin E (50 mg/d) and vitamin C (150 mg/d) for 6 months reduced plasma oxidative damage and enhanced the erythrocyte activities of CAT and GR (Ferrer et al., 2010). Therefore, the use of topical vitamin C for the improvement of patients with skin symptoms remains to be studied.

### Atopic Dermatitis

Atopic dermatitis (AD) is a chronic relapsing inflammation of the skin associated with allergies. The lesions are characterized by erythematous papules with itching or scaling, affecting 15–30% of children (Bieber, 2008; Kim et al., 2013; Tollefson and Bruckner, 2014). One reason this is important is the structural or functional damage of the skin barrier (Sivaranjani, 2013; Zaniboni et al., 2016). Keratinocytes and their intercellular lipids are important components of the human skin barrier, and vitamin C can promote keratinocyte differentiation and the production of interstitial material (Savini et al., 2002; Kim et al., 2011). As the most abundant lipid in the skin barrier material, ceramide is generated at the end of keratinocyte differentiation (Uchida et al., 2001). AD patients lack several nutrients, including vitamin A and vitamin C. A greater number of food allergens have shown an association with an increase in the number of deficient nutrients (Gorman et al., 2007). The ratio of vitamin C intake is significantly higher in more than three restricted groups compared to the non-restricted group, which demonstrates that vitamin C can improve chronic inflammation and positively influence AD and that the intake of several foods containing high levels of vitamin C and vitamin A may be related to a decrease in the risk of AD and asthma diseases (Cook et al., 1997; Forastiere et al., 2000; Arora et al., 2002; Lim et al., 2013; Park and Zippin, 2014). Vitamin C can stimulate ceramide production in keratinocytes and improve overall epidermal barrier function (Kim K.P. et al., 2015). With increases in clinical symptoms of AD, vitamin C and ceramide levels were reduced, which demonstrated that vitamin C and ceramide levels and the severity of AD are positively correlated (Shin et al., 2016). Although vitamin C can be an adjuvant treatment for a variety of dermatitises, oral vitamin C still causes symmetrical AD (Assier et al., 2013).

### Malignant Melanoma

Melanoma derived from melanocytes is a kind of skin tumor that is more malignant and occurs in the skin, skin and mucous membrane transfer and removal of the eye choroid (Yussif et al., 2017). Vitamin C may have an effect on the function and quantity of melanocytes, thereby reducing the synthesis of melanocytes (Kameyama et al., 1996). The antimelanogenesis effect of vitamin C is mainly due to its role as a reducing agent in the various oxidation stages of melanin formation (Choi et al., 2010b). Vitamin C can indirectly inhibit the activity of tyrosinase because of its antioxidant capacity, thus reducing melanogenesis. Furthermore, vitamin C can also reduce the melanogenesis of melanoma cells stimulated by α-melanocyte-stimulating hormone (α-MSH) *in vitro* (Stojkovic-Filipovic and Kittler, 2014). However, whether this has an effect in the clinical treatment of melanoma has not yet been determined. Moreover, cancer patients have been shown to have very low reserves of ascorbic acid, which is essential for the structural integrity of the intercellular matrix. Degradation of the extracellular matrix correlates with the aggressiveness of tumor growth and invasiveness of a cancer. Vitamin C supplementation significantly reduced the metastasis of B16FO melanoma in Gulo knockout (KO) mice and inhibited the growth of 4T1 breast cancer cells in scorbutic mice (Cha et al., 2013). Surgical resection is effective only for non-metastatic, early tumors, and there is still not a good curative chemotherapy for patients with tumor metastasis (Hill et al., 2015; Kaminska-Winciorek et al., 2015), although vitamin C has an inhibitory effect on the invasion and metastasis of melanoma (Bedogni and Powell, 2009; Miles et al., 2015). Vitamin C can reduce tumor growth, invasion and metastasis of melanoma in mice by inhibiting the hypoxia inducible factor-1 alpha (HIF-1α) transcriptional activity, which might play a key role in melanoma carcinogenesis (Cha et al., 2011; Miles et al., 2015). Posttranslational regulation of HIF-1α relies on proline hydrogenase and the inhibition of HIF hydrogenase, both of which require ascorbate as a cofactor (Cha et al., 2011). The toxic effects of vitamin C on tumor cells may be related to the induction of oxidative stress in cells. However, when the antioxidation system of tumor cells is incomplete, the balance is destroyed, and the oxygen-promoting effect of vitamin C leads to the death of tumor cells (Kim K. et al., 2015; Uetaki et al., 2015). Vitamin C is often used as an adjunct to chemotherapy for tumors. Vitamin C can also increase 5-hydroxymethylcytosine (5hmC) content in melanoma cells and cause a decrease in tumor-cell invasiveness and growth (Gustafson et al., 2015). Thus, vitamin C can be regarded as a potential antitumor drug for the prevention of invasion and metastasis of melanoma, which weakens the tumor capsule integrity and invasiveness and reduces the degree of malignancy.

However, there is still a lack of understanding about the route of administration for vitamin C, the dosage of medication and the complications. We should increase awareness of the fact that high concentrations of vitamin C induce apoptosis of malignant melanoma cells, while low concentrations promote the growth of tumor cells (Yang et al., 2017). However, it is worth noting that the toxic effects of vitamin C on cancer cells are valid only with intravenous administration and not in cases of oral administration (Levine et al., 2011). An increase in vitamin C levels in the diet of white women increased the risk of melanoma, also demonstrating that only intravenous vitamin C increased plasma ascorbic acid concentration and that oral preparation had no effect on plasma concentration (Feskanich et al., 2003; Ohno et al., 2009).

### Herpes Zoster and Postherpetic Neuralgia

Herpes zoster and its sequelae, postherpetic neuralgia, are conditions with significant morbidity. HZ is caused by the reactivation of latent varicella zoster virus (VZV) that lurks in the body and classically affects adults older than 50 years old (Insinga et al., 2005; Weaver, 2009). The specific clinical manifestations are clustered, small blisters distributed along the unilateral peripheral nerves with acute neuralgia (Nair et al., 2014). PHN refers to the persistence of neuralgia 4 weeks after the disappearance of herpes lesions and is a chronic, debilitating neuropathic pain that can persist long beyond the resolution of visible cutaneous manifestations (Nalamachu and Morley-Forster, 2012; Gan et al., 2013; Marrero et al., 2017). Given the different pathogeneses of HZ and PHN, the symptoms can be divided into stimulus-induced pain and spontaneous pain. Spontaneous pain can be persistent or intermittent (paroxysmal). Stimulation-induced pain is often classified as mechanical, thermal, or chemical (Chen et al., 2009). Recent studies have proposed that this pain is related to the participation of oxygen free radicals. Peripheral inflammation stimulates nociceptors to produce oxygen free radicals. Oxygen free radicals participate in the stimulation of pain after they accumulate in the body (Kim et al., 2004). Vitamin C, as an oxidant, has been reported to have a clinical analgesic effect (Carr and McCall, 2017). In addition, the incidence of PHN in patients with HZ who lack plasma vitamin C has been significantly higher than the incidence in patients with normal plasma vitamin C levels. When vitamin C supplementation is given to patients with HZ, the probability of subsequent PHN in those patients is greatly reduced, demonstrating that vitamin C has a preventive effect on PHN (Chen et al., 2011). A clinical case report also mentions that intravenous injection of vitamin C immediately relieves pain in HZ patients and related symptoms in PHN patients. In addition, vitamin C can be fully used as a therapeutic adjuvant for patients who are resistant to analgesics (Schencking et al., 2010; Byun and Jeon, 2011). Kim et al. (2016) found that clinical administration of vitamin C supplementation cannot alleviate the immediate severe pain caused by HZ but has a better preventive effect on clinical symptoms caused by PHN (Kim et al., 2016). In addition, vitamin C directly affects the immune system to reduce the chance of viral infection in the body (Hemilä, 2017), similar to the application of vitamin D, which can affect the immune mechanisms of the human body (Chao et al., 2015). Therefore, whether the combined use of vitamin C and vitamin D has a good and comprehensive therapeutic effect on the presence of HZ or PHN is still a question worth exploring.

### Other Diseases

Vitamin C in other dermatological diseases is seen as an adjuvant for use in combination with other drugs or for physical therapy. It has good therapeutic potential in a variety of dermatological diseases, such as acne, allergic contact dermatitis, psoriasis, and progressive purpura, especially when used in combination with other clinical drugs (**Table 2**). *Propionibacterium acne* (*P. acne*) plays an active pro-inflammatory role in the whole process of acne and is involved in the skin keratinocytes and sebaceous glands of the pilosebaceous follicle, resulting in the generation of acne (Beylot et al., 2014). The combination of zinc and clarithromycin, along with vitamin C, has an antibacterial effect against clarithromycin-resistant *P. acnes in vitro* (Iinuma et al., 2011), providing a new idea for the clinical use of antibiotics in the treatment of acne bacteria. Vitamin C, combined with microneedle treatment for acne scars, improved skin hardness, smoothness, and postinflammatory pigmentation (Chawla, 2014). Vitamin C can also reduce allergies often encountered in dermatology. A case report concluded that it reduced the allergic contact dermatitis caused by hair dyes (Basketter et al., 2016). The main component of hair dye is *p*-phenylenediamine. Skin pretreated with vitamin C can inhibit the allergic reaction induced by *p*-phenylenediamine (Coenraads et al., 2016). More interestingly, in an experimental study on the role of LXR-a (liver X receptor alpha) in the pathogenesis of psoriasis, Soodgupta et al. (2014) demonstrated that ascorbic acid and atorvastatin combined with 22-*r*-hydroxycholine returned female hormone-treated psoriatic keratinocytes to normal. Collagen is an important component in the connective tissue of the basement membrane and capillary vessels, and vitamin C is essential for collagen synthesis. Although the pathogenesis of progressive pigmented purpuric dermatosis remains unclear, there are hypotheses that it is associated with increased vascular fragility (Sardana et al., 2004). Increased vascular fragility is associated with reduced collagen in vascular connective tissue. The use of a combined therapy of aloin and vitamin C also has a good effect on progressive pigmented purpuric dermatosis (Schober et al., 2014). Regarding vitamin C as an adjuvant for physical therapy, it has been reported that oral pidotimod and vitamin C can be combined after laser vaporization for the treatment of female genital herpes, which can improve immunity and natural defenses and reduce the persistence of HPV infection (Zervoudis et al., 2010). Supplementing patients with high-dose vitamin C significantly improved the treatment effect of short-term UVB irradiation treatment in patients with vitiligo, especially in the UVB-irradiated skin area (Don et al., 2006). However, patients with renal insufficiency, deficiency in glucose 6-phosphate dehydrogenase or paroxysmal nocturnal hemoglobinuria should not use vitamin C because vitamin C can cause poisoning (Padayatty et al., 2010).

**Table 2:** The roles of vitamin C as a therapeutic adjuvant in other skin diseases.

Skin disease	The roles of vitamin C	Combined drugs/physical therapy	Reference
Acne	Against clarithromycin-resistant *P. acnes*	Zinc and clarithromycin	Iinuma et al., 2011; Beylot et al., 2014;
Acne scars	Improve skin hardness, smoothness, and postinflammatory pigmentation	Microneedle treatment	Chawla, 2014
Allergic contact dermatitis	Reduces the elicitation reaction to a *p*-phenylenediamine (PPD)-containing hair dye	Pretreatment of the skin with the antioxidant ascorbic acid	Basketter et al., 2016; Coenraads et al., 2016
Psoriatic	Make keratinocytes return to normal	Atorvastatin combined with 22-*r*-hydroxycholine female hormone-treated	Soodgupta et al., 2014
Progressive pigmented purpuric dermatosis (PPPD)	Protect blood vessel collagen, reduce vascular fragility, prevent disease recurrence	Aloin	Sardana et al., 2004; Schober et al., 2014
Genital herpes	Improve immunity and natural defenses and reduce the persistence of HPV infection	Vaporization laser treatment, pidotimod	Zervoudis et al., 2010
Vitiligo	Increase hyperpigmentation at pigment diminished spots	Short-term UVB irradiation treatment	Don et al., 2006


## Conclusion

In conclusion, nutritional strategies suggest the potential benefits of a diet rich in vitamin C as a preventive tool for patients with skin diseases. Vitamin C has low toxicity, is easy to obtain, and has a low price. Therefore, if it can be applied to clinical treatment in dermatology, the prospects should be very impressive. Notably, vitamin C supplementation modulated inflammatory cytokine secretion, decreased metastasis of melanoma, reduced tumor growth and enhanced the encapsulation of tumors resulting from a breast cancer challenge. Following these studies, investigation into the impact of excessive food limitations on growth, malnutrition, and skin disease management is needed, and further studies should investigate the wide and effective therapeutic potential of vitamin C in dermatology. Although ascorbate supplementation in cancer patients has been proposed to reverse their scorbutic symptoms and treat their cancer, dermatologists should take into consideration the potential risks of the clinical use of vitamin C to minimize the risk of treatment. In addition, the route of administration for the use of vitamin C should receive more attention. It is necessary to increase the concentration of vitamin C in peripheral blood intravenously to be toxic to tumor cells. Since vitamin C is a water-soluble molecule and its transdermal absorption efficiency is low, it may be of great significance to identify efficient transdermal drug delivery methods for the stabilization of active compounds, such as finding lipophilic derivatives of vitamin C to increase the absorption through the epidermis. Thus, the clinical use of vitamin C in patients with skin diseases still requires caution.

## Author Contributions

HL contributed the conception. KW, HJ, WL, MQ, and TD analyzed the data. KW and HJ wrote the manuscript. HL and KW revised the manuscript.

## Conflict of Interest Statement

The authors declare that the research was conducted in the absence of any commercial or financial relationships that could be construed as a potential conflict of interest.
